# Pulmonary COVID-19: Learning Spatiotemporal Features Combining CNN and LSTM Networks for Lung Ultrasound Video Classification

**DOI:** 10.3390/s21165486

**Published:** 2021-08-14

**Authors:** Bruno Barros, Paulo Lacerda, Célio Albuquerque, Aura Conci

**Affiliations:** Institute of Computing, Campus Praia Vermelha, Fluminense Federal University, Niterói 24.210-346, Brazil; placerda@id.uff.br (P.L.); celioalbuquerque@id.uff.br (C.A.); aconci@id.uff.br (A.C.)

**Keywords:** COVID-19, CNN, Deep Learning, LSTM, lung ultrasound, neural networks, hyperparameter optimization

## Abstract

Deep Learning is a very active and important area for building Computer-Aided Diagnosis (CAD) applications. This work aims to present a hybrid model to classify lung ultrasound (LUS) videos captured by convex transducers to diagnose COVID-19. A Convolutional Neural Network (CNN) performed the extraction of spatial features, and the temporal dependence was learned using a Long Short-Term Memory (LSTM). Different types of convolutional architectures were used for feature extraction. The hybrid model (CNN-LSTM) hyperparameters were optimized using the Optuna framework. The best hybrid model was composed of an Xception pre-trained on ImageNet and an LSTM containing 512 units, configured with a dropout rate of 0.4, two fully connected layers containing 1024 neurons each, and a sequence of 20 frames in the input layer (20×2018). The model presented an average accuracy of 93% and sensitivity of 97% for COVID-19, outperforming models based purely on spatial approaches. Furthermore, feature extraction using transfer learning with models pre-trained on ImageNet provided comparable results to models pre-trained on LUS images. The results corroborate with other studies showing that this model for LUS classification can be an important tool in the fight against COVID-19 and other lung diseases.

## 1. Introduction

Since the first cases of COVID-19 were registered in a hospital in Wuhan City, China [1,2] in December 2019, more than 122 million confirmed cases and 2.7 million deaths worldwide have been reported to the World Health Organization (WHO) [3]. Currently, the disease continues to spread, and new virus variants, some of them more resistant to antibody neutralization, are appearing rapidly. Additionally, these variants have a higher transmission rate [4,5,6], leading to the appearance of new epidemic outbreaks in various parts of the globe [7], potentially overloading the entire health system.

COVID-19 transmission is difficult to control. It depends on many aspects, being the object of study by several researchers who have proposed different artificial intelligence (AI) techniques to forecast the spread [8,9,10,11] and aid in the medical diagnosis of COVID-19 [12,13,14]. Other studies have considered the impact of climatic and urban variables on the forecast models for the spread of the disease [15,16]. In addition to significant lifestyle changes in large cities, COVID-19 has also impacted the economy and financial markets, as investigated in [17]. These works reflect the rapid effort of science to understand the impact caused by the disease and reinforce the importance of AI as a powerful tool in the fight against COVID-19.

Studies based on patients affected by COVID-19 indicate a high prevalence of respiratory symptoms that require a comprehensive evaluation [18,19]. The standard method for diagnosing the disease involves a clinical examination, performing pulmonary auscultation of the patient using a stethoscope, and then additional imaging examinations such as X-ray (XR) and computed tomography (CT) [20,21]. The laboratory test known as reverse transcription-polymerase chain reaction (RT-PCR) is considered the gold standard [22] being used to diagnosis the disease. However, this test, in addition to demanding time (about two days), presents results that indicate a low sensitivity of 70%, according to [23], making a result confirmation necessary.

Clinical and imaging exams are prone to the spread of the virus, mainly due to the number of people in the medical team mobilized to examine and move the patient to the centers where the diagnostic imaging equipment is located. Another aspect that must be considered is the possibility of the virus spreading through the contamination of equipment and of the stethoscope itself, which needs to be sterilized at each examination [20]. In addition to the risk of contamination, there is the fact that the stethoscope and auscultation in these cases are of low proven usefulness.

Studies indicate that thorax ultrasound can surpass the current standard of care, both in speed and diagnosis in cases of respiratory failure [24]. Lung ultrasound (LUS) has good sensitivity in detecting lung pathology (bacterial or viral) [25,26]. Regarding COVID-19, studies report a high correlation between the clinical findings of the LUS and the chest CT examination [27,28,29,30,31,32]. In this sense, some studies suggest the use of ultrasound as an alternative to auscultation and auxiliary XR and CT exams [33,34,35,36,37].

Ultrasound (US) has advantages that can help fight COVID-19, as it is portable, radiation-free, easy to sterilize, has a low acquisition cost, allows the examination to be performed at the bedside, and can be used by the physician without the need to mobilize other professionals [38]. Moreover, due to the portability and low cost of US equipment, such devices enable rapid prototyping of systems that make intensive use of AI and computer vision (CV) techniques to aid in the diagnosis of COVID-19 [39]. However, despite the advantages presented in the use of LUS images for the diagnosis of pulmonary pathology and COVID-19, few studies are exploring Deep Learning (DL) techniques compared to the number of studies performed with other medical imaging techniques such as XR and CT [21,40,41,42,43].

DL models are currently considered state-of-the-art in many CV and medical applications. According to the review conducted in [44], many applications surpass or equal the results obtained by human specialists. Although the use of DL has advanced in the medical field, including CAD [45,46,47], the application of these techniques in US images can be considered incipient [48]. Considering the main global issue since the last year, the COVID-19 pandemic, there are works on DL related with promising results, which corroborates the clinical findings in the area.

These techniques are predominantly applied to other types of medical imaging (XR and CT) [21,49,50,51,52,53,54,55,56,57,58,59], while studies that consider US imaging in their medical diagnostic models are less frequent. One of the possible causes for it is the lag of public databases available for scientific research, especially concerning LUS videos containing proved cases of COVID-19, according to a survey conducted by [60]. Another possible cause may be that some have a concern over US studies’ quality since it is a modality dependent on the skill of the operator [61].

In the review performed with five studies and 466 participants [62], despite the US having a good sensitivity of 86.4% for the diagnosis of COVID-19, the specificity presented in the results was 54.6%; that is, the ability to diagnose healthy individuals is low. There is an opportunity for DL experiments to improve the ability of human experts to diagnose positive and negative cases of COVID-19.

**Figure 1:**
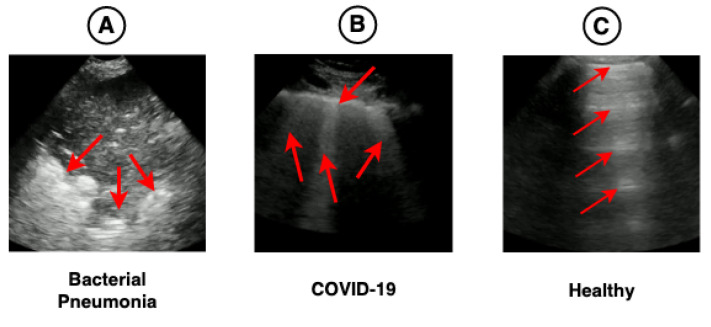
Image (**A**) shows a large consolidation area related to the diagnoses of bacterial pneumonia. Image (**B**) shows B-lines (vertical lines) and an irregular pleura line, both related to lung ultrasound (LUS) diagnosis of COVID-19. Image (**C**) is a lung with a healthy diagnosis, where there are A-lines (horizontal lines) and a regular pleural line. Red arrows were manually added to show the location of pathologies in the images.

In this sense, this work proposes a hybrid model (CNN-LSTM) of video classification to aid in diagnosing COVID-19, using spatial and temporal features present in 185 LUS videos. Furthermore, this research investigates the impact of using different pre-trained CNN architectures on ImageNet and LUS images to extract spatial features. Finally, we used a technique known as Gradient-weighted Class Activation Mapping (Grad-CAM) capable of providing visual explanations about the most important parts of the images to verify whether these highlighted regions are related to the clinical findings of COVID-19, as shown in Figure 1.

## 2. Theoretical Background

### 2.1. Deep Learning

One of the problems with Artificial Neural Networks (ANN) is the limitation of learning capacity. An ANN with only one layer restricts the number and complexity of functions that the optimization process can learn. Thus, depending on the complexity of the data, it can be difficult to separate the feature space in classes since it is not possible to separate them linearly in most real cases.

For cases where space is not linearly separable, the ANN architecture can be modified to learn more complex functions and break this restriction of data linearity. According to [63], the implementation of two intermediate layers makes the approximation of any function possible, which makes deep neural networks (DNN) a more attractive option.

As the network gets deeper, it is noticed that the learning capacity increases and the adjustment of the function $f^{\prime }$ gets better and better. Each neuron in the initial layer learns a function that defines a hyperplane capable of separating subspaces of a given space. In this way, a neuron from a subsequent layer combines the group of hyperplanes learned by neurons from the previous layers forming convex regions. In the last layer, we have the combination of these convex regions in regions of arbitrary shape [64].

This architecture can capture more complex functions; this ends up introducing other problems, such as overfitting and a demand for higher computational power. Due to the advancement in computational power provided by graphic processing units (GPU), which are now at increasingly affordable costs, it has become possible to adopt models based on DL in medical diagnosis, and they have been growing more and more in the industry [65]. Consequently, AI techniques such as DL have become very active and vital tools for building computer-aided diagnosis (CAD) systems [66].

#### 2.1.1. Convolution Neural Networks

Convolution Neural Networks (CNNs) are a specific class of deep neural networks (DNN) that is closely linked to the area of CV due to the operation of convolution included in some of their layers. Initially, CNNs were proposed for image classification problems [67]. However, currently, CNNs have different types of applications such as object detection, image segmentation, synthetic image generation, among other types of applications in the medical field [68,69].

Due to their great performance in classical CV applications, this class of DNN quickly ended up becoming state of the art [70]. The architecture of a CNN favors the learning of spatial patterns using the convolution layers. The idea behind convolutions in DNN is almost the same as in CV; i.e., to filter the image content in order to enhance its features. When successive convolutions are applied along with the layers of the network, it is possible to extract increasingly complex features. In contrast, the cardinality of the feature set of the images decreases [71].

Unlike structured tables and other input data, images present spatial patterns that can be learned and reused. The features that are learned from the images are invariant. An edge detection operation, for example, can be easily be reused in different parts of the processing. Furthermore, due to convolution operations, network connections become sparse; that is, each output depends only on a small number of input parameters [72]. This hierarchical construction reduces the number of parameters involved in training, which is a tremendous advantage over fully connected neural networks.

Many CNN architectures have been developed in the last two decades. Each one presents different configurations, which makes a definition of the best configuration for a given application a big challenge. They vary in terms of the number of layers, layer types, size of convolutional filters, operations, connections between layers, amount of memory used by the network, and number of parameters involved in training, among other aspects [73,74]. Consequently, it is important to apply the proper architecture for each type of problem. VGG [75], Resnet [76], Densenet [77], InceptionV3 [78], InceptionResNetV2 [79], Xception [80], MobileNet [81], MobileNetV2 [82], and EfficientNet [83] are some architectures widely applied for diagnosis based on images.

#### 2.1.2. Recurrent Neural Networks

Recurrent neural networks (RNNs) were introduced in 1980 as networks specialized in time series data. They are a class of DNN inspired by the concept of biological memory [84], where historical information is used in computation and the weights are shared over time. RNNs process data sequentially for both input and output.

Unlike CNNs, RNNs can process sequences of different dimensions as input. CNNs do not have memory, only transmitting data from the input layer towards the output, disregarding temporal dependencies.

The recurrent term in RNNs comes from the fact that the layers work in a loop. The calculations in time *t* depend on the calculations performed previously, that is, at $t_{-1}$, $t_{-2}$, $t_{-3}$, …, $t_{-n}$ [85]. Although this class of DNN solves problems related to time series and time dependence very well, other problems end up emerging, so new architectures need to be investigated to solve them. For instance, one of the most well-known problems in RNNs is the fact that they suffer from the vanishing gradients, caused by updating the network weights, which, when considering a large input time interval $\left(t_{i}\right)$ or sequence, causes the network to become very deep [86,87].

#### 2.1.3. Long Short-Term Memory

In the problem known as vanishing gradients, when backpropagation through hidden layers occurs, the gradients tend to become infinitesimally small, and at this point, updating the network weights in these cases ends up being insignificant, preventing the weights from changing, making the learning of very long sequences a difficult task to accomplish [86]. To avoid this problem, it is recommended to change the initialization of network weights and activation functions and use new network architectures designed for this purpose, such as Long Short-Term Memory (LSTM) [86,88,89] and Gated Recurring Unit (GRU) [90].

GRUs are a more recent class of RNN and have a performance often comparable to the results obtained by LSTMs. They are simpler and more computationally efficient, but the performance of both types of networks ends up being dependent on the dataset, which makes choosing RNN a trial and error process [91].

LSTMs can be considered a generalization of GRUs and can work with long time series, considering information in more distant times. This class of RNN favors work with long sequences. This is why LSTMs have been applied to different areas of knowledge, including medical diagnosis, being frequently used in problems involving video classification and recognition of human activities [92,93,94,95].

LSTM and GRU use the concept of gates, which aim to regulate the flow of information. In this way, gates learn which sequence of information must be kept or discarded. In GRUs, there are two types of gates: the reset gate and the update gate. These gates allow the recurrent network to control what information should be passed to the output. In this way, gates can be trained to maintain information from distant times and remove information irrelevant to prediction. GRUs have only one state known as a hidden state. This state is transferred among the time steps being responsible for maintaining the short- and long-term dependencies at the same time.

While GRUs have only one state, LSTMs have two different states transmitted among cells: the cell state and the hidden state. They carry long and short-term memory, respectively. In addition to this difference, LSTMs have three gates: forget gate, input gate, and output gate. The forget gate controls the information to be discarded on the cell state. The input gate controls the addition of valuable information to the cell state. The output gate controls the extraction of valuable information from the cell state to the output.

#### 2.1.4. Transfer Learning

Transfer learning (TL) is an effective strategy for training a new model on a small dataset. In this technique, the network is pre-trained on a large dataset, such as ImageNet [96], to then be reused and applied to a task that has few available data, taking advantage of the learned features [97]. The ImageNet database has millions of images and hundreds of annotated object categories. Its creation was aimed at facilitating the training of DL models focusing on image classification, object location, and object detection tasks [98].

As discussed earlier, there are different DL models pre-trained on the ImageNet dataset available for use. These models come with training weights, which makes them easy to reuse in similar problems. Therefore, it is possible to take advantage of state-of-the-art models, i.e., reuse of architectures recently published by the scientific community, transferring the learning of generic features to other domains, such as medical image classification.

**Figure 2:**
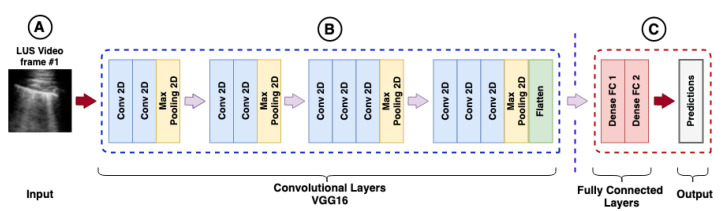
Example of feature extraction using a VGG16: (**A**) represents the input layer of VGG16 (224×244×3). (**B**) represents the convolutional base, with convolutional layers, pooling, and flatten operations (convolutional layer output). (**C**) represents the fully connected layers, followed by the prediction layer (output layer). The layers represented in (**C**) need to be removed for the convolutional neural network (CNN) to work as a feature extractor.

The extraction of features from a CNN is considered a powerful technique [99], where TL is used to provide to new networks features learned in another dataset. In this sense, the dense layers of a pre-trained CNN are removed, and the remaining layers, called the convolutional base, are kept, as shown in Figure 2. The idea is that the preserved part works as a feature extractor. That, is the layers represented in Figure 2C need to be removed for the CNN to work as a feature extractor. In this way, it is possible to extract only the features without any classification and use them in other models, such as RNNs.

The most common form of TL is adding one or more dense layers to the convolutional base, which has its weights frozen while additional dense layers are trained using a small relevant dataset. In this process, only the weights of the added layers are learned, taking advantage of the weights frozen from the convolutional layers, decreasing considerably the number of parameters to be trained. Another form of TL is fine-tuning. The training weights of the convolutional base are unfrozen, or even the entire model weights and training is performed with a low learning rate. Thus, the initial weights provided by the pre-trained model are used, adapting the learning to the new data [85].

#### 2.1.5. Overfitting

Overfitting occurs when a DNN perfectly learns the weights based on the training set but cannot adequately capture the process that generated this data. Therefore, when new results are evaluated based on test set, they are not as good as those obtained in training set.

In DL, overfitting can occur in cases where the training set has insufficient data. Consequently, the model cannot successfully generalize and predict unseen instances of the problem in the training process. To improve generalization in DNN, it is necessary to increase the number of data, reduce the complexity of the model, or apply regularization techniques such as those mentioned in Section 2.1.6 and Section 2.1.7.

#### 2.1.6. Dropout

In the regularization technique known as Dropout [100], there is no addition of the regularization term in the cost function. Unlike L1 and L2 regularization [101], in the Dropout technique, a modification occurs directly in the network architecture in such a way that some hidden layer connections are randomly discarded with a probability *p* configured in training (usually between 0.1 and 0.5 ).

#### 2.1.7. Batch Normalization

In the technique known as Batch Normalization [102] the change in internal covariation and instability in the distributions of the network activations is reduced, applying a transformation that keeps the average output close to 0 and the standard deviation close to 1, contributing to making the network more stable, accelerating training and combating overfitting.

#### 2.1.8. Pooling

Pooling acts on the convolutional layer as an operator, just like convolution. The role of pooling is to serve as an aggregation operation, which aims to summarize output feature maps. There are no parameters to be trained in pooling. However, its importance lies in the fact that this type of operation reduces the sensitivity of convolutional layers to the effect of location and spatially reduced representations [103].

As with the convolutional layer, the pooling operation uses a kernel that reduces the size of the image representation or feature map, just as in convolution. A low-resolution representation is created in the pooling operation, keeping the significant structural elements but discarding the fine details.

The most used types of pooling are average pooling and maximum pooling. In the first case, the result is the average of the elements superimposed on the kernel and, in the second case, the maximum element. The kernel will be given as the reference window; the average and the maximum will be calculated based on the pixels of the reference image or input feature map. The result will be a low-resolution image and a model less susceptible to the effects of affine transformation (as rotation, displacement, and translation) [104].

#### 2.1.9. Flatten

Flatten is an operation used to transform the output of a layer into a one-dimensional vector. This flattening works like a DNN standard input. In this way, the result of this flattening can be transferred to a dense layer. For example, in a pooling operation with 20 representations of an small image, for instance, a matrix (3×3), or a 2D array, this is performed with a tensor (or a 3D array) of size (3×3×20), but when a flattening operation is applied, such a result will be represented as a one-dimensional array (or a 1D vector) with size equal to 180.

#### 2.1.10. Grad-CAM

Gradient-Weighted Class Activation Mapping (Grad-CAM) is a technique for producing visual explanations in a CNN-based model [105]. Grad-CAM uses gradients to create a location map that highlights the most important regions of the image. The idea is to use these regions to explain the decision-making model.

### 2.2. Hyperparameter Optimization

The hyperparameter optimization (HPO) problem can be characterized by searching for optimal hyperparameters that maximize or minimize the result of an objective function. Therefore, the optimization process tries to find the best set of hyperparameters that maximizes or minimizes a relevant metric for the problem domain.

The two most used methods are Grid Search and Random Search. In the Grid Search, the search is performed exhaustively using all combinations of hyperparameters in the search space. In the Random Search, the search is performed randomly and, therefore, with lower computational cost than the search performed in the first method without guaranteeing an optimal result.

Searching the entire search space running all models and their different combinations of hyperparameters can take a considerable amount of time given the high amount of hyperparameters involved in training [106]. The hyperparameter is an aspect of the search space and is part of the model configuration; unlike a network parameter, it cannot be automatically learned in the training process.

Hyperparameters influence the training result, consequently, on the evaluation metrics themselves. However, due to the difficulty of performing the optimization considering the search in the entire search grid (Grid Search), other methods emerged, mainly focused on improving the search for hyperparameters, making the process more efficient.

Bayesian Optimization [107,108,109] is a method known as Black-Box and is based on a probability model, where the method attempts to learn a costly objective function based on previous observations. Some known implementations are: SMAC [110], HYPEROPTO [111], MOE [112], and pyGPGO [113]. As opposed to Bayesian optimization, the methods known as Multi Fidelity try to estimate the objective function in a cheaper way. Among these Multi Fidelity methods, three approaches are highlighted: (1) Successive Halving [114], (2) Hyperband [115], and (3) BOHB [116]. The Successive Halving method attempts to cut in half the number of models tested in an iteration, discarding those configurations of hyperparameters that did not produce good results and keeping the other half until only one winning model remains. However, there is a conflict between the number of hyperparameter settings that must be selected and the number of cuts required. Thus, Hyperband is a proposal to extend the Successive Halving method and aims to solve this trade-off, proposing to frequently perform the Successive Halving method with different budgets [115]. The BOHB method is an efficient combination between Hyperband and Bayesian Optimization. It is a flexible, scalable, and robust method; however, it also depends on the budget [116].

## 3. Related Work

Currently, the use of CNNs applied to diagnosing diseases already achieves performances comparable to human experts. Some works related to DL in the medical field have shown great potential and even outperform the metrics achieved by human experts. Among these works, the following applications are highlighted: screening for diabetic retinopathy [117], classification of skin lesions [118], detection of lymph node metastases [119], and classification of pneumonia [120].

Some studies were carried out with a focus on ultrasound images. In [121], the authors proposed a CNN trained on short ultrasound videos of pigs with controlled pulmonary conditions to detect five features related to various types of pulmonary diseases: B-lines, merged B-lines, lack of lung sliding, consolidation, and pleural effusion. A total of 2200 LUS videos were collected from 110 exams. The proposed model reached at least 85% sensitivity for these all diseases except for B-lines and 86% specificity.

A CNN was tested to classify the presence of B-lines based on the LUS of a dataset containing 400 videos referring to emergency patients [122]. The proposed model presented a sensitivity of 93% and specificity of 96% for the classification of B-lines (0–1) [122].

In [39], the authors presented an efficient and lightweight network called Mini- COVIDNet based on MobileNets, with a focus on mobile devices. The network was trained on LUS images to classify them into three classes: bacterial pneumonia, COVID-19, and healthy. As a result, the model was able to achieve an accuracy of 83% (best result). The total training time was 24 min, with a size of 51.29 MB, requiring fewer parameters in its configuration.

From data collected at the Royal Melbourne Hospital, 623 videos of LUS, containing 99,209 ultrasound images of 70 patients were used. In addition, a DL model using a Spatial Transformer Network (STN) for the automatic detection of pleural effusion focusing on COVID-19 was proposed in [123]. The model was trained using supervised and weakly supervised approaches. Both approaches presented an accuracy above 90%, respectively (92% and 91%).

In [124], a hybrid architecture was proposed that integrates a CNN and an LSTM to predict the severity score of COVID-19 (4 scores) based on 60 LUS videos (39 from convex transducers and 21 from linear transducers) from 29 patients. The result of the proposed model was an average accuracy of 79% for the linear transducers and 68% for the convex transducers.

A CNN model based on STNs was proposed to predict the four disease severity scores and the location of pathological artifacts in a weakly supervised way in [125]. For the experiments, a dataset containing 277 LUS videos from 35 patients was used, corresponding to a total of 58,924 images, where 45,560 recorded from convex transducers and 13,364 acquired using linear transducers, distributed as follows: 19,973 score 0 (34%), 14,295 score 1 (24%), 18,972 score 2 (32%), 5684 score 3 (10%). The frame-based prediction result for the F1-score was 65% and for video-based prediction 61%. In segmentation, the result presented an accuracy of 96% and a Dice score of 75%. In [52], the authors performed a study with different types of CNNs suggesting a VGG19 for classification into three classes: bacterial pneumonia, COVID-19, and healthy. The study considered XR, CT, and LUS images from datasets of different publicly available sources. This work highlights the performance of the trained model in LUS images, reaching an accuracy of 86% for training with XR, 100% for ultrasound, and 84% for CT.

Three types of CNNs (VGG-19, Resnet-101, and EfficientNet-B5) for the classification of LUS images according to eight types of clinical stages were used in [126]. The dataset consisted of 10,350 images collected from different sources. The results showed that the CNN based on the EfficientNet-B5 architecture outperformed the others, presenting on average for classification into the eight types of clinical stages: F1-score 82%, accuracy 95%, sensitivity 82%, specificity 97%, and precision 82%. In addition, other results were presented based on the grouping types (three and four) where the average accuracy was 96% (three types) and 95% (four types).

A CNN model that uses multi-layer feature fusion for the classification of COVID-19 was presented in [127]. The model was trained based on LUS images from convex transducers. A total of 121 videos were used, 23 with a diagnosis of bacterial pneumonia, 45 with a diagnosis of COVID-19, and 53 with a healthy diagnosis. The proposed method obtained a precision of 93% and an accuracy of 92%.

LUS images with the presence of B-lines of different etiologies were used for training a CNN in [128]. For training, 612 videos (12,1381 images) of B-lines were used, referring to 243 patients classified into three classes: Acute Respiratory Distress Syndrome (ARDS), COVID-19, and Hydrostatic Pulmonary Edema (HPE). The result obtained showed an ability to discriminate between the three proposed classes, being the area under the receiver operator characteristic (ROC) curve (AUC) achieved: for ARDS 93%, COVID-19 100%, and HPE 100%.

In [129], the authors proposed a frame-based model for video classification using an ImageNet pre-trained VGG16 in LUS videos. The dataset consisted of 179 videos and 53 images (convex transducers) totaling 3234 frames, where 704 frames belonged to the bacterial pneumonia class, 1204 belonged to the COVID-19 class, and 1326 frames to the healthy class. The model resulted in a mean accuracy of 90%, a sensitivity of 90%, a precision of 92%, an F1-score of 91%, and specificity of 96% for COVID-19.

## 4. Materials and Methods

In addition to a complete description of the approach used, the input data and the hybrid model proposed in this work were provided to readers, making this article reproducible. The data can be viewed in the open source repository https://github.com/b-mandelbrot/pulmonary-covid19 (accessed on 9 August 2021).

### 4.1. Ultrasound Devices

In this work, we used videos captured with a low-frequency convex transducer. They present low resolution but are more suitable for LUS used at the bedside environment. In addition, they have a longer acoustic wavelength and provide better penetration and visualization of deeper structures. This type of transducer is, therefore, best suited for evaluating consolidations and pleural effusions [27,130]. On the other hand, high-frequency transducers have a high resolution compared to low-frequency transducers and are more suitable for evaluating the pleural region.

As described in Section 4.2, due to the nature of the data source, it was impossible to verify details about the manufacturer and model of the ultrasound devices used to capture the videos, except for 20 videos belonging to the manufacturer Butterfly, representing approximately 11% of available videos.

### 4.2. Building the LUS Dataset

The data set used in this study was constructed based on LUS videos made publicly available by different sources, such as hospitals, medical companies, and scientific publications. This dataset was selected, validated by medical experts, and published in [129]. The download of data can be performed automatically. The videos were pre-processed, with rulers and other artifacts removed using the scripts provided with the dataset, facilitating the construction of training and test data as described in the article.

In our work, 185 videos captured by convex transducers referring to 131 patients were considered. Videos captured by linear transducers were discarded (22 videos). In addition, videos of viral pneumonia were not included because there were only three videos in such class. A summary of the number of used videos per class is presented in Table 1.

**Figure 3:**
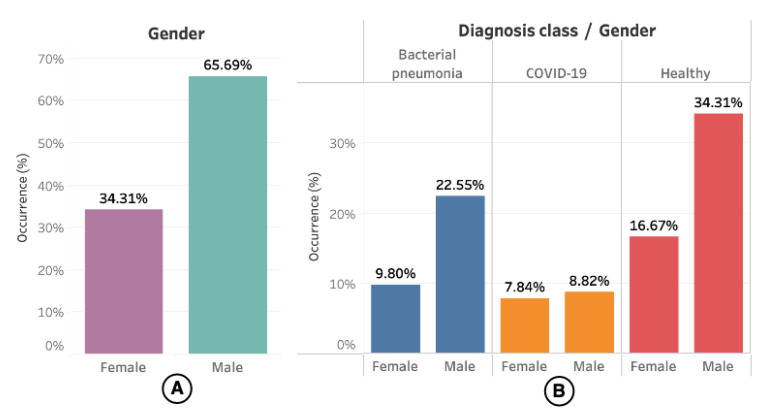
(**A**) shows the occurrence of gender in the LUS videos. (**B**) shows the occurrence of gender segmented by diagnosis class.

Regarding demographic data, only 67 (51.14%) of them had information about sex and age available. The gender distribution concerning the videos (or US exams) can be seen in Figure 3, where 102 (55.13%) videos had information about the patient’s gender.

**Figure 4:**
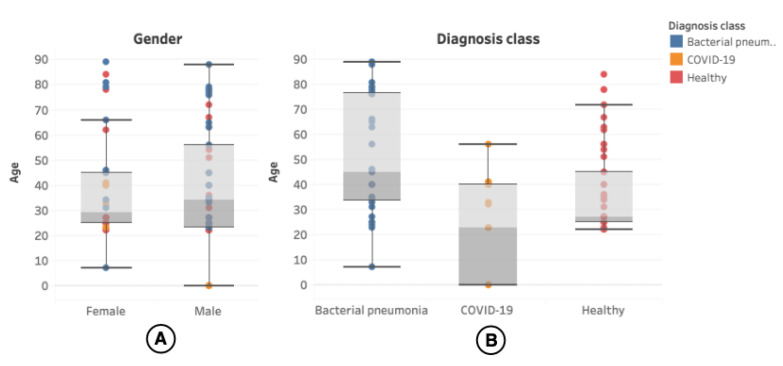
(**A**) shows the distribution of age in the LUS videos. (**B**) shows the distribution of age segmented by diagnosis class. Each colored dot on the image represents a patient undergoing the LUS exam and the different diagnosis classes to which the exam is related.

The patient’s age was present in 100 (54.05%) of the videos, and the distribution can be seen in more detail in Figure 4.

**Figure 5:**
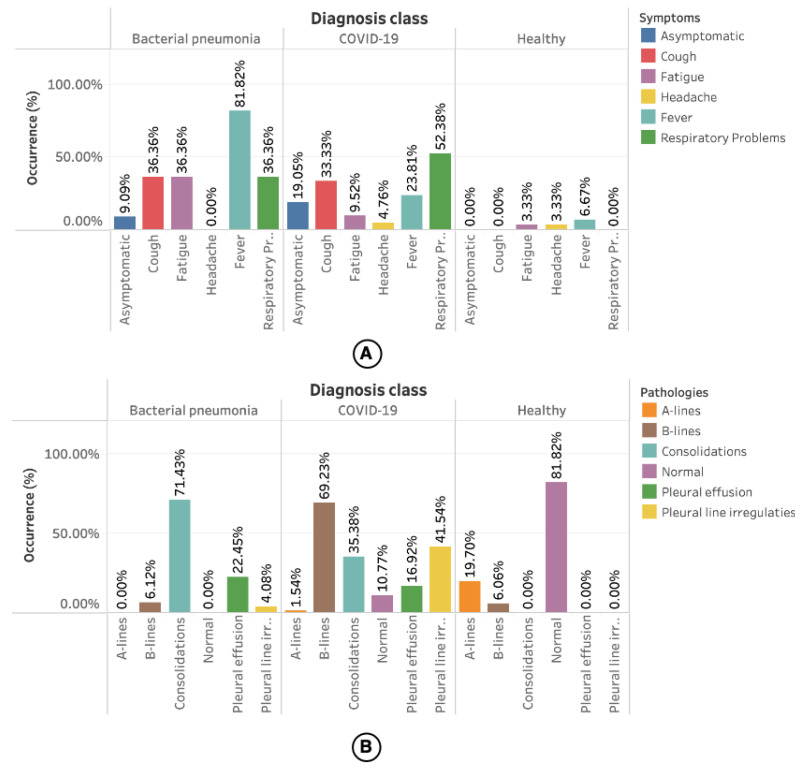
(**A**) shows the occurrence of symptoms in the LUS videos segmented by diagnosis class. (**B**) shows the occurrence of pathologies segmented by diagnosis class.

The statistics related to the symptoms presented by the patients and the pathologies related to the LUS videos are presented in Figure 5. However, only 34% of the videos had information about the symptoms.

Figure 5A shows the occurrence of symptoms by diagnosis class. Most of the symptoms reported for bacterial pneumonia are fever (81.82%). For COVID-19, 52.38% of cases are respiratory problems. In healthy patients, a minimum portion had symptoms such as fatigue (3.33%), headache (3.33%), and fever (6.67%), but without any relationship with the diseases mentioned earlier.

Information about the pathologies found in the videos was available for all videos used in this work. As shown in Figure 5B, most occurrences for bacterial pneumonia are consolidation (71.43%) followed by pleural effusions (22.45%). Among the pathologies reported for COVID-19 are B-lines (69.23%), followed by pleural line irregularities (41.45%). A-lines (19.70%) followed by a smaller number of B-lines (6.06%) were reported in the videos referring to healthy patients.

### 4.3. Cross Validation (K-Folds)

To verify the generalization capacity of the optimized models, the K-Folds cross-validation technique was used, in which the dataset is divided into *k* partitions [131]. The training used k−1 partitions, and the test the remaining partition k−4.

The study used k=5 partitions and each model was evaluated for each partition. The final result was expressed by the weighted sum of the model’s performance in each partition. The objective was to balance the results, as the models were trained on all partitions and were also tested on each of the remaining partitions (the last one out from the training data). Therefore, this technique helps combat model overfitting, as it attempts to balance the results using each partition.

The five partitions were stratified according to the distribution of classes, ensuring that all partitions had the same original distribution and there was no overlap of patients between the training and testing partitions. For compatibility of results, we use the open source script provided with the dataset. After executing the script for the partitioning of the data, we obtained the result presented in Table 2.

### 4.4. Video Processing

The OpenCV library available for the Python language was used to analyze the number of frames available in the videos. Statistics can be viewed in Table 3. Each of the 185 videos was processed, and the image related to each frame of the video was extracted and normalized. For the extraction of frames, we adopted a maximum limit equal to the minimum number of available frames: 21 frames.

**Figure 6:**
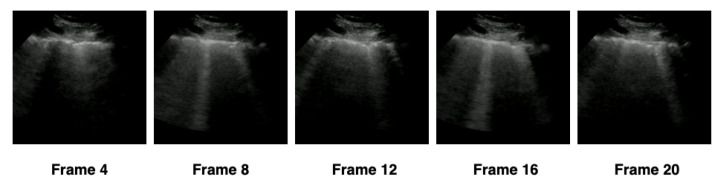
Samples of how the 5 frames were considered on configuration 1 of Table 4. The frames belong to a video whose patient was diagnosed with COVID-19.

In order to verify the ideal number of frames that the hybrid model should use, we separated the extraction into four configurations—(1) 5 frames, (2) 10 frames, (3) 15 frames, and (4) 20 frames—according to Table 4. The frames were extracted at constant intervals, based on the number of frames in each configuration. We adopted as a minimum limit the value of 5 frames and the maximum limit of 20 frames, standardizing the settings in intervals of 5 frames. For example, in the first configuration, a video containing 21 frames would have 5 frames extracted with an interval of 4 frames between them, and 16 frames would be discarded, as represented in Figure 6. The total number of frames extracted by each configuration was presented in Table 4.

For each of the configurations, the respective frames of each video were extracted. Next, the pixel values of the images were scaled, where the value of each pixel was multiplied by the factor of (1/255). Finally, images were resized using the nearest interpolation algorithm to a fixed size of (224×244) with 3 RGB channels to maintain compatibility with the pre-trained models on ImageNet.

### 4.5. Feature Extraction with CNNs

For the extraction of features, different CNN architectures were used according to Section 2.1.1. The purpose of CNNs was to capture the spatial features of the sequence of images belonging to each video.

In this work, pre-trained models in LUS images containing the diagnosis of COVID-19 provided in [129] (POCOVID-Net) were used. Furthermore, models based on transfer learning were used with CNNs pre-trained on ImageNet, without any prior training in medical images, as explained in Section 2.1.4.

The frames extracted from the videos based on each configuration of Table 4 were submitted to CNNs proposed in this investigation to extract the main features of the three classes of interest: bacterial pneumonia, COVID-19, and healthy. As the networks were already pre-trained, it was not necessary to retrain the CNNs or perform any fine-tuning, for both pre-trained on LUS images or for the models pre-trained on ImageNet. These features were represented by one-dimension vectors extracted from the convolutional layers located in the deeper layers of the networks. Finally, the dense layers were removed as shown in Figure 2.

Table 5 summarizes the CNN architectures used in our work and the length of the one-dimension vector extracted from the respective convolutional layers. Once these features have been extracted from the frames, they are now ready to be used as input to an LSTM.

### 4.6. Training Temporal Data with LSTMs

**Figure 7:**
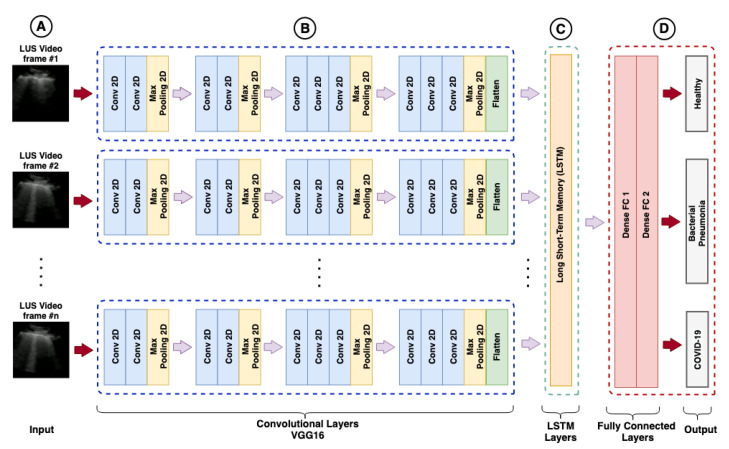
Hybrid model (VGG16-LSTM): Column (**A**) represents the sequence of images extracted from the videos. Group (**B**) represents the convolutional layers based of a VGG16. Group (**C**) represents the long short-term memory (LSTM) layer responsible for learning temporal features. Group (**D**) represents the classification layer.

The training of recurrent models aimed to classify the LUS videos, using as input a sequence of features extracted from the images by different CNN models and as output the prediction of the three classes of interest, as shown in Figure 7.

The RNN training depends on the used CNN architecture and the features configuration. The input layer of RNN models has a different number of elements both for features and numbers of frames used in the video sequence analysis, as shown in Table 6. Each sequence represents the input layer of an LSTM.

The features extracted by CNNs are part of spatial learning. Each sequence of *n* frames based on extraction settings and flat feature vectors is used as input to an RNN model. An LSTM for each CNN architecture was trained under sets of hyperparameters tested and varied using an optimization framework.

### 4.7. Hyperparameter Optimization with Optuna

The training was performed using a framework called Optuna [132], available as a library for the Python language. Optuna is an open-source software that easily allows the construction of organized experiments, besides providing several algorithms for sampler and pruner. It is possible to run the experiments in a distributed way, and the results are visualized in a web interface (dashboard).

For the sampler, the Tree-structured Parzen Estimator (TPE) algorithm was used [111] and for pruner, the Hyperband [115] of Section 2.2 was adopted. A total of 68 models were optimized, each model corresponded to feature specific configurations and a CNN architecture (4×17). Finally, each model was submitted to 100 trials.

The LSTM architecture was fixed on a single LSTM layer, with two dense layers fully connected with the ReLU activation function before the prediction layer. In addition, the prediction layer (output layer) was configured with the cross-entropy categorical loss function. The parameters chosen for optimization were: (1) the number of units used in the LSTM; (2) the dropout rate; (3) the number of neurons in the fully connected layers; (4) the learning rate (LR), and the batch size to be used in training.

The Adam optimizer was adopted as the standard for training the models, and no other optimizers were used. The optimization process was carried out considering the accuracy of the models in the five partitions, according to Section 4.3. The average accuracy was used as the value returned by the objective function to be maximized, and the best models were saved so that their accuracies could be compared.

Keras and TensorFlow Python libraries were used to build the CNN and RNN models. The optimization and training process was performed on an NVIDIA DGX-1 machine composed of eight Tesla P-100 GPUs containing 16 GB of memory for each GPU. However, for this experiment, only 1 GPU was used. The optimization and training process took ≈64 h. A summary of the values obtained from the hyperparameters by the optimization process can be found in Table 7.

Table 8 lists all the values referring to the metrics of the models evaluated after the optimization process ordered by accuracy and F1-score (COVID-19) presented in the first and last columns. Although the table lists values with two decimal places of precision, we consider more decimal places in case of ties, as available in https://github.com/b-mandelbrot/pulmonary-covid19/blob/master/evaluation.csv (accessed on 9 August 2021).

### 4.8. Visual Explanations with Grad-CAM

**Figure 8:**
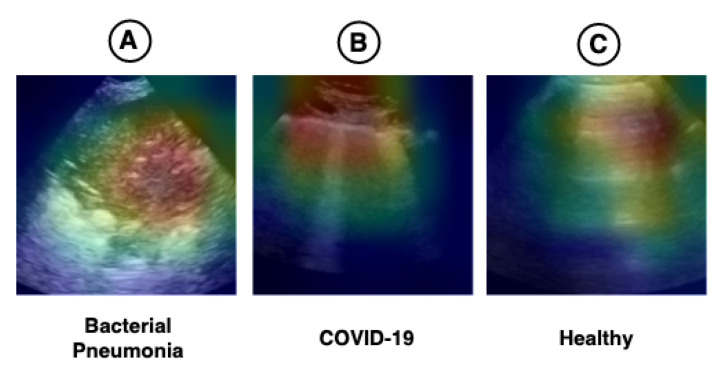
Heat maps referring to the regions considered the most important for the model (Xception) according to the Grad-CAM technique. Image (**A**) shows a large consolidation area related to the diagnoses of bacterial pneumonia. Image (**B**) shows B-lines (vertical lines) and an irregular pleura line, both related to US diagnosis of COVID-19. Image (**C**) is a lung with a healthy diagnosis, where there are the named A-lines (horizontal lines) and a regular pleural line.

The hybrid model (CNN-LSTM) proposed in this work has an Xception in its convolutional base. Therefore, this technique cannot be fully explained, but the part of the model used to extract spatial features (CNN) can be visualized using this technique. We applied Grad-CAM to verify whether the regions highlighted in the images have any relationship with the pathologies found in the LUS videos. The result can be seen in Figure 8.

Figure 1 shows the main pathologies found in the LUS videos regarding the diagnoses of bacterial pneumonia, COVID-19, and healthy. Figure 8A demonstrates that the region of consolidations is highlighted in the heat map. In Figure 8B, the highlighted regions are coherent with the pathologies known as B-lines and pleural line irregularities present in COVID-19 images. In Figure 8C, the heat map shows the A-lines, which are found in LUS exams referring to healthy patients. The heat maps presented in Figure 8 lead us to believe that the model can use the features learned in images outside the application domain (i.e., medical images), such as those from ImageNet, for use in LUS images.

## 5. Discussion

This work presents a new hybrid model for classifying LUS videos for the diagnosis of COVID-19. The LUS video classification proposed involved two types of data: spatial data, referring to video frames, and temporal data, represented by time-indexed frames. The extraction of spatial features was performed by a CNN, and the temporal dependence between video frames was learned using an LSTM.

In this work, 68 hybrid models (CNN-LSTM) were trained to classify the videos. Each model was composed of a different CNN architecture. The frames referring to the videos were extracted in four configurations (5, 10, 15, and 20 frames). In addition, two types of pre-trained models were tested, the POCOVID-Net [129], pre-trained on LUS images, and 12 CNN architectures, pre-trained on the ImageNet dataset. Each of these models went through an HPO process, where the best results were stored for comparison, as shown in Table 7. In order to balance the results and prevent the models from overfitting, the cross-validation technique was used. The average accuracy was used as the value of the objective function to be maximized.

The number of frames used by each model and its hyperparameters varied according to the available configurations. All extraction configurations provided good results, but only two models optimized with the five-frame configuration were between the top 10 best hybrid models. All other models showed better results when using configurations with more than five frames. Regarding the extraction of spatial features, both ImageNet and LUS pre-trained architectures obtained good results. The best model was pre-trained on ImageNet and used a 20-frame extraction configuration.

**Figure A1:**
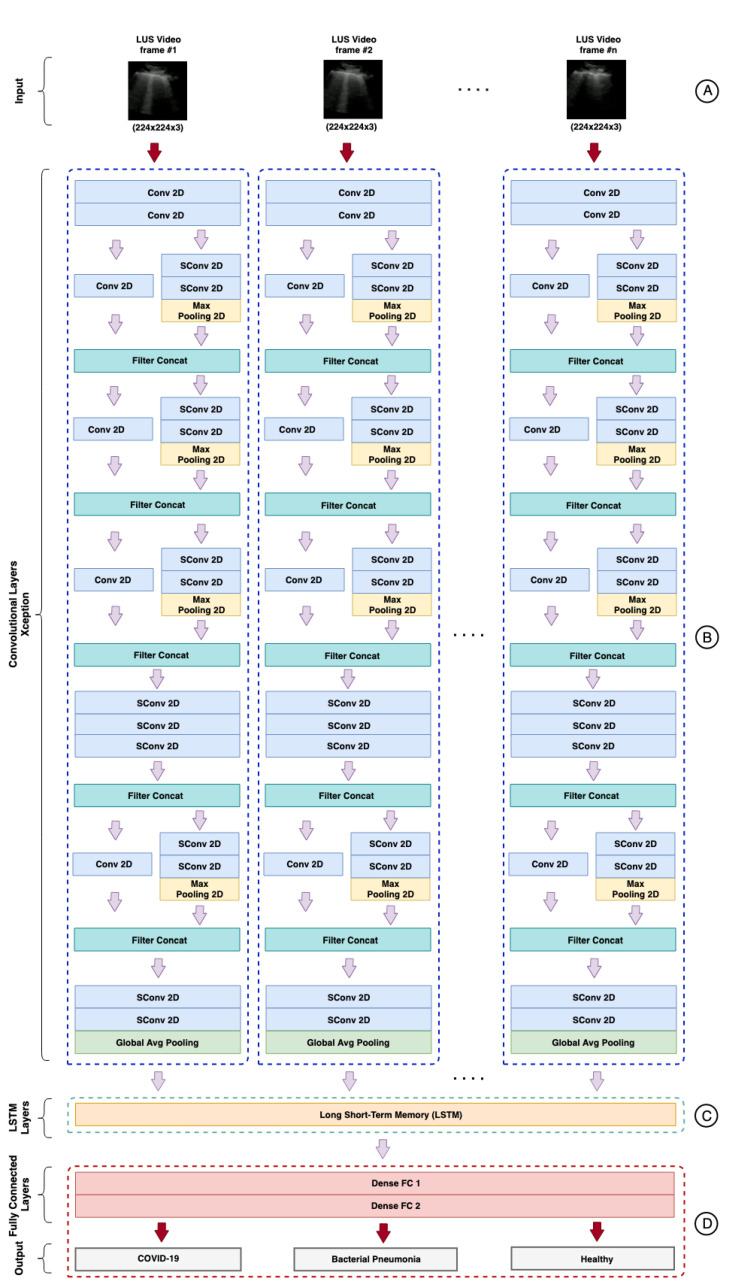
Hybrid model (Xception-LSTM): (**A**) represents the sequence of frames extracted from the videos. (**B**) represents the convolutional layers, the figure shows the convolutional base of an Xception. (**C**) represents the LSTM layer responsible for learning temporal features. (**D**) represents the classification layer.

According to the results obtained and presented in Table 8, the best hybrid model was the Xception-LSTM, composed of a pre-trained Xception on ImageNet and an LSTM containing 512 units, configured with a dropout rate of 0.4 and a sequence of 20 frames in the input layer (20×2018). The architecture proposed for the hybrid model can be visualized in Figure A1, Appendix A.

Regarding the numeric results, the best model presented an average accuracy of 0.93±0.13, precision of 0.94±0.12, sensitivity of 0.97±0.06, specificity of 0.96±0.09, and F1-Score of 0.95±0.10 for COVID-19. The POCOVID-Net-3-LSTM model whose convolutional base is pre-trained on LUS images obtained a result very close to the Xception-LSTM pre-trained on ImageNET, an average accuracy of 0.92±0.17, precision of 0.91±0.18, sensitivity of 0.97±0.06, specificity of 0.92±0.16, and F1-Score of 0.93±0.13 for COVID-19.

This spatiotemporal model outperformed the purely spatial version in all metrics except precision and specificity, with values of 0.91±0.18 and 0.92±0.16 versus 0.92±0.07 and 0.96±0.04 of the spatial model [129]. Furthermore, the spatiotemporal version has fewer parameters (837 K) than its spatial version (14.7 M). In this sense, we highlight the NASNetMobile-LSTM that obtained an accuracy of 0.92±0.17 with the smallest number of parameters among the top 10 models, as seen in the Table 7.

All models in the top 10 presented results superior to those obtained by human experts according to the study carried out in [62], where the reported combined sensitivity was 86.4%, and the specificity was 54.6%. Other architectures achieved similar results, such as DenseNet121, DenseNet201, NASNetLarge, and Resnet152V2.

## 6. Conclusions

The results indicate that the use of hybrid models (CNN-LSTM) can be effective in learning spatiotemporal features, exceeding the performance of models with purely spatial approaches [39,129] and even human experts [62]. However, these results should be interpreted with parsimony. Few data are available on the performance of human experts in LUS imaging for the diagnosis of COVID-19.

Transfer learning with models pre-trained on ImageNet provided comparable results to models pre-trained on LUS images, suggesting that ImageNet can be used in cases where there is limited data for training [99]. We also show that the use of transfer learning techniques and HPO can facilitate the creation of rapid prototypes for diagnosing diseases, as seen in other studies [21].

This study provided evidence that the LUS-based imaging technique can be an essential tool in containing COVID-19 and other lung diseases such as bacterial pneumonia. However, there is still room for further experiments.

As future work, it is intended to increase the number of LUS videos, adding new sources so that it is possible to test the models with independent videos. In addition, we plan to carry out new tests with other types of RNNs, such as GRUs. As mentioned in Section 2.1.3, GRUs are more efficient and, depending on the dataset, can provide results comparable to or even better than LSTMs.

## Figures and Tables

**Table 1:** Data of convex transducer US videos included in this work.

Diagnosis Class	Videos	Percentage of Total
Bacterial Pneumonia	50	27%
COVID-19	69	37%
Healthy	66	36%
Total	185	100%

**Table 2:** Distribution of classes in 5 partitions separated by training and testing.

Fold Number	Train/Test	COVID-19	Bacterial Pneumonia	Healthy	Total
0	Train	56	40	53	149
0	Test	13	10	13	36
1	Train	56	40	53	149
1	Test	13	10	13	36
2	Train	56	40	52	148
2	Test	13	10	14	37
3	Train	55	40	53	148
3	Test	14	10	13	37
4	Train	53	40	53	146
4	Test	16	10	13	39

**Table 3:** Data related to the number of frames per video.

Statistics	Number of Frames
Minimum	21
Median	111
Mean	148
Maximum	458
Standard Deviation	100

**Table 4:** Number of frames extracted per configuration.

Configuration	Frames per Video	Number of Extracted Frames
1	5	925
2	10	1850
3	15	2775
4	20	3700

**Table 5:** Elements of feature vector.

CNN Architecture	Elements of Feature Vector
DenseNet121	1024
DenseNet169	1664
DenseNet201	1920
EfficientNetB0	1280
InceptionResNetV2	1536
MobileNetV2	1280
NASNetLarge	4032
NASNetMobile	1056
POCOVID-Net 1, 2, 3, 4, 5	512
ResNet152V2	2048
VGG16	25,088
VGG19	25,088
Xception	2048

**Table 6:** Characteristics of used sequences.

CNN Architecture	LSTM Input 1	LSTM Input 2	LSTM Input 3	LSTM Input 4
DenseNet121	5×1024	10×1024	15×1024	20×1024
DenseNet169	5×1664	10×1664	15×1664	20×1664
DenseNet201	5×1920	10×1920	15×1920	20×1920
EfficientNetB0	5×1280	10×1280	15×1280	20×1280
InceptionResNetV2	5×1536	10×1536	15×1536	20×1536
MobileNetV2	5×1280	10×1280	15×1280	20×1280
NASNetLarge	5×4032	10×4032	15×4032	20×4032
NASNetMobile	5×1056	10×1056	15×1056	20×1056
POCOVID-Net 1, 2, 3, 4, 5	5×512	10×512	15×512	20×512
ResNet152V2	5×2048	10×2048	15×2048	20×2048
VGG16	5 × 25,088	10 × 25,088	15 × 25,088	20 × 25,088
VGG19	5 × 25,088	10 × 25,088	15 × 25,088	20 × 25,088
Xception	5×2048	10×2048	15×2048	20×2048

**Table 7:** Summary of the hyperparameters of the top 10 best hybrid models.

Hybrid Model	Input Layer	LSTM Units	LSTM Dropout	FC Layer 1	FC Layer 2	Learning Rate	Batch Size	Total Params
Xception-LSTM	20×2048	512	0.4	1024	1024	$6.55\times 10^{-4}$	8	6,822,915
NASNetLarge-LSTM	20×4032	1024	0.5	64	256	$6.62\times 10^{-4}$	12	20,796,483
DenseNet121-LSTM	20×1024	32	0.1	1024	1024	$9.08\times 10^{-4}$	20	1,221,763
POCOVID-Net-3-LSTM	5×512	256	0.2	128	128	$8.18\times 10^{-4}$	32	837,251
DenseNet201-LSTM	5×1920	1024	0.2	512	128	$2.54\times 10^{-4}$	4	12,653,571
NASNetMobile-LSTM	15×1056	64	0.5	64	1024	$6.60\times 10^{-4}$	12	360,771
POCOVID-Net-1-LSTM	20×512	256	0.2	256	512	$5.21\times 10^{-4}$	32	986,371
POCOVID-Net-4-LSTM	10×512	512	0.1	128	512	$1.63\times 10^{-3}$	24	2,232,451
ResNet152V2-LSTM	10×2048	1024	0	1024	128	$7.76\times 10^{-6}$	4	13,768,195
ResNet152V2-LSTM	20×2048	512	0.1	128	256	$1.18\times 10^{-5}$	8	5,344,387

**Table 8:** Summary of the top 10 models ordered by average accuracy and F1-Score for COVID-19.

Hybrid Model	Class	Precision	Recall	Specificity	F1-Score
Xception-LSTM	Bacterial Pneumonia	0.92 ± 0.17	0.94 ± 0.12	0.96 ± 0.08	0.93 ± 0.15
(20 frames)	COVID-19	0.94 ± 0.12	0.97 ± 0.06	0.96 ± 0.09	0.95 ± 0.10
Acc. 0.93 ± 0.13	Healthy	0.95 ± 0.10	0.89 ± 0.22	0.98 ± 0.03	0.91 ± 0.17
NASNetLarge-LSTM	Bacterial Pneumonia	0.91 ± 0.17	0.86 ± 0.23	0.98 ± 0.05	0.88 ± 0.21
(20 frames)	COVID-19	0.91 ± 0.14	0.94 ± 0.12	0.95 ± 0.08	0.93 ± 0.13
Acc. 0.92 ± 0.14	Healthy	0.93 ± 0.13	0.95 ± 0.09	0.96 ± 0.09	0.94 ± 0.11
DenseNet121-LSTM	Bacterial Pneumonia	0.94 ± 0.11	0.90 ± 0.20	0.98 ± 0.03	0.92 ± 0.16
(20 frames)	COVID-19	0.92 ± 0.15	0.95 ± 0.09	0.95 ± 0.10	0.94 ± 0.12
Acc. 0.92 ± 0.16	Healthy	0.91 ± 0.18	0.91 ± 0.18	0.95 ± 0.10	0.91 ± 0.18
POCOVID-Net-3-LSTM	Bacterial Pneumonia	0.93 ± 0.13	0.88 ± 0.24	0.98 ± 0.03	0.90 ± 0.20
(5 frames )	COVID-19	0.91 ± 0.18	0.97 ± 0.06	0.92 ± 0.16	0.93 ± 0.13
Acc. 0.92 ± 0.17	Healthy	0.92 ± 0.16	0.89 ± 0.22	0.97 ± 0.07	0.90 ± 0.19
DenseNet201-LSTM	Bacterial Pneumonia	0.89 ± 0.22	0.90 ± 0.20	0.95 ± 0.09	0.90 ± 0.21
(5 frames)	COVID-19	0.93 ± 0.13	0.92 ± 0.15	0.97 ± 0.07	0.93 ± 0.14
Acc. 0.92 ± 0.17	Healthy	0.92 ± 0.15	0.92 ± 0.15	0.96 ± 0.09	0.92 ± 0.15
NASNetMobile-LSTM	Bacterial Pneumonia	0.93 ± 0.13	0.84 ± 0.32	0.99 ± 0.02	0.86 ± 0.28
(15 frames)	COVID-19	0.90 ± 0.20	0.97 ± 0.06	0.90 ± 0.19	0.93 ± 0.15
Acc. 0.92 ± 0.17	Healthy	0.95 ± 0.11	0.92 ± 0.15	0.97 ± 0.05	0.93 ± 0.13
POCOVID-Net-1-LSTM	Bacterial Pneumonia	1.00 ± 0.00	0.88 ± 0.24	1.00 ± 0.00	0.91 ± 0.17
(20 frames)	COVID-19	0.92 ± 0.16	0.89 ± 0.22	0.97 ± 0.07	0.90 ± 0.19
Acc. 0.92 ± 0.17	Healthy	0.90 ± 0.20	0.97 ± 0.06	0.90 ± 0.19	0.93 ± 0.15
POCOVID-Net-4-LSTM	Bacterial Pneumonia	0.92 ± 0.16	0.92 ± 0.16	0.97 ± 0.06	0.92 ± 0.16
(10 frames)	COVID-19	0.91 ± 0.18	0.89 ± 0.22	0.96 ± 0.09	0.90 ± 0.20
Acc. 0.92 ± 0.17	Healthy	0.92 ± 0.16	0.94 ± 0.12	0.95 ± 0.10	0.93 ± 0.14
ResNet152V2-LSTM	Bacterial Pneumonia	0.93 ± 0.13	0.84 ± 0.32	0.99 ± 0.02	0.86 ± 0.28
(10 frames)	COVID-19	0.91 ± 0.17	0.92 ± 0.15	0.95 ± 0.10	0.92 ± 0.16
Acc. 0.91 ± 0.18	Healthy	0.91 ± 0.19	0.95 ± 0.09	0.92 ± 0.16	0.92 ± 0.15
ResNet152V2-LSTM	Bacterial Pneumonia	0.89 ± 0.23	0.86 ± 0.28	0.97 ± 0.06	0.87 ± 0.26
(20 frames)	COVID-19	0.91 ± 0.18	0.91 ± 0.18	0.95 ± 0.10	0.91 ± 0.18
Acc. 0.91 ± 0.18	Healthy	0.92 ± 0.15	0.95 ± 0.09	0.95 ± 0.10	0.94 ± 0.12

## Data Availability

Publicly available datasets from lung ultrasound were analyzed in this study. This data can be found here: https://github.com/jannisborn/covid19_ultrasound (accessed on 25 February 2021).

## Abbreviations

The following abbreviations are used in this manuscript:

AI	Artificial Intelligence
ANN	Artificial Neural Networks
ARDS	Acute Respiratory Distress Syndrome
AUC	Area Under Curve
CAD	Computer Aided Diagnosis
CNN	Convolutional Neural Networks
CT	Computed Tomography
CV	Computer Vision
DL	Deep Learning
DNN	Deep Neural Networks
GPU	Graphic Processing Unit
GRU	Gated Recurrent Units
Grad-CAM	Gradient-weighted Class Activation Mapping
HPE	Hydrostatic Pulmonary Edema
HPO	Hyperparameter Optimization
LSTM	Long Short-Term Memory
LUS	Lung Ultrasound
PPE	Personal Protective Equipment.
RNN	Recurrent Neural Networks
ROC	Receiver Operator Characteristic
STN	Spatial Transformer Networks
US	Ultrasound
XR	X-ray

## Appendix A

This appendix presents the proposed architecture of the hybrid model in Figure A1, an Xception pre-trained on ImageNet, and an LSTM containing 512 units, configured with a dropout rate of 0.4, two fully connected layers containing 1024 neurons each, and a sequence of 20 frames in the input layer (20×2018).
